# Enhanced Charge Separation in Nanoporous BiVO4 by External Electron Transport Layer Boosts Solar Water Splitting

**DOI:** 10.1002/advs.202305567

**Published:** 2023-12-07

**Authors:** Xiaotian Yang, Jianpeng Cui, Luxue Lin, Ang Bian, Jun Dai, Wei Du, Shiying Guo, Jingguo Hu, Xiaoyong Xu

**Affiliations:** ^1^ College of Physics Science and Technology, and Interdisciplinary Research Center Yangzhou University Yangzhou 225002 China; ^2^ School of Science Jiangsu University of Science and Technology Zhenjiang 212100 China

**Keywords:** BiVO_4_ photoande, charge transport, photochemical cell, solar water splitting

## Abstract

The optimization of charge transport with electron‐hole separation directed toward specific redox reactions is a crucial mission for artificial photosynthesis. Bismuth vanadate (BiVO_4_, BVO) is a popular photoanode material for solar water splitting, but it faces tricky challenges in poor charge separation due to its modest charge transport properties. Here, a concept of the external electron transport layer (ETL) is first proposed and demonstrated its effectiveness in suppressing the charge recombination both in bulk and at surface. Specifically, a conformal carbon capsulation applied on BVO enables a remarkable increase in the charge separation efficiency, thanks to its critical roles in passivating surface charge‐trapping sites and building external conductance channels. Through decorated with an oxygen evolution catalyst to accelerate surface charge transfer, the carbon‐encased BVO (BVO@C) photoanode manifests durable water splitting over 120 h with a high current density of 5.9 mA cm^−2^ at 1.23 V versus the reversible hydrogen electrode (RHE) under 1 sun irradiation (100 mW cm^−2^, AM 1.5 G), which is an activity‐stability trade‐off record for single BVO light absorber. This work opens up a new avenue to steer charge separation via external ETL for solar fuel conversion.

## Graphical abstract

X. Yang et al. proposed a concept of external electron transport layer and demonstrated its practicality to improve charge separation by using the carbon envelope on BiVO_4_ nanoparticles as an interconnect network for electron extraction and transport. This report opens up a new avenue to address the poor bulk charge transport that embarrasses most oxide semiconductors for designing PEC systems.

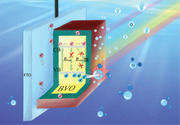

## 1 Introduction

Photoelectrochemical (PEC) water splitting has attracted enormous interest as a promising technology that can directly convert solar energy to hydrogen fuel for renewable solar storage and utilization.^[^ ^1^, ^2^, ^3^ ^]^ Bismuth vanadate (BiVO_4_, BVO) has emerged as one of the most promising photoanode materials for solar water splitting since it has an appropriate bandgap (2.4,2.5 eV) in response to visible light and a deep valance band potential competent for water oxidation.^[^ ^4^, ^5^, ^6^, ^7^, ^8^, ^9^, ^10^ ^]^ However, PEC performance achieved with BVO to date has been far below what is predicted in theory, mainly suffering from serious charge recombination occurring at surface and in bulk due to poor intrinsic charge transport properties (e.g., short hole diffusion length of 70–100 nm and low electron mobility of 0.02–0.044 cm^2^ V^−1^ s^−1^ in polycrystalline BVO).^[^ ^11^, ^12^ ^]^ To this end, engineering morphologies and facets,^[^ ^13^, ^14^, ^15^ ^]^ introducing dopants or defects,^[^ ^16^, ^17^, ^18^, ^19^ ^]^ constructing heterostructures,^[^ ^20^, ^21^, ^22^ ^]^ and decorating with oxygen evolution catalysts (OECs)^[^ ^23^, ^24^, ^25^, ^26^ ^]^ have been identified as effective strategies of improving charge transport and separation to enhance PEC performance of BVO photoanodes. Among these techniques, heterojunction engineering with electron transfer layers (ETLs)^[^ ^27^, ^28^ ^]^ or hole transfer layers (HTLs)^[^ ^21^, ^22^ ^]^ promises to steer charge migration with targeted separation, which is highly desirable for PEC water splitting.

The BVO/WO_3_ heterojunction has emerged as the most typical example using the ETL to facilitate electron transport and block hole transfer due to the type‐II band alignment and superior electron mobility along WO_3_.^[^ ^29^, ^30^ ^]^ Park et al. employed various WO_3_ nanostructures as conductive scaffolds underneath to allow more BVO loading for maximizing the type‐II interface effect.^[^ ^31^, ^32^, ^33^ ^]^ As a result, an impressive photocurrent density of 5.34 mA cm^−2^ at 1.23 V versus the reversible hydrogen electrode (RHE) was realized using the Mo‐doped BVO/WO_3_ helix heterostructure decorated with a NiOOH/FeOOH cocatalyst.^[^ ^32^ ^]^ Several other materials with suitable bandgaps, high electron mobility, and diffusion length, such as SnO_2_, TiO_2,_ and ZnO, have also been used as promising ETLs for BVO,^[^ ^34^, ^35^, ^36^ ^]^ in which the underlying type‐II junctions at heterointerfaces were responsible for the cascaded electron migration from BVO to base collector.

Note that ETLs based on type‐II junctions must be buried between BVO absorber and base collector; moreover, only BVO/ETL heterointerfaces help to improve electron extraction and block hole transfer (i.e., the so‐called hole mirror). So, the role of ETLs in navigating electron transport surely suffers from the incrassated BVO overlayer, but the sufficient optical thickness is needed for light harvesting. This contradiction remains a critical factor limiting apparent photocurrent density yield at BVO photoanodes.^[^ ^8^, ^37^ ^]^ Particularly in a porous film composed of nanoparticles (NPs), the large number of internal contact junctions severely impede electron transport especially as the thickness increases, although its large specific surface allows more OEC loading to support surface catalysis. The thickness of porous BVO NP film with optimum performance is generally only 300–400 nm,^[^ ^6^, ^38^ ^]^ which is far insufficient for optical absorption. Therefore, an effective regulation on bulk electron transport to break through the obstacle caused by the thickened absorber layer is highly expected for improving the PEC performance, while no viable solution is reported yet.

In this work, we for the first time propose an external ETL concept via using carbon outerwear encasing porous BVO as electron transport network. We encapsulate the photosensitive BVO NPs with ultrathin carbon outerwear (BVO@C) through a biological template self‐assembly process to form a 3D porous architecture with interconnected conductive enclosure. The conformal carbon coating passivates surface charge‐trapping sites and forms 3D cascaded electron channels, enabling a valuable ability to improve electron transport against bulk and surface recombination. The BVO@C photoanode thus affords near‐complete charge separation at 1.23 V_RHE_ even though absorber layer 811 nm thick enough for optical absorption. In coordination with an OEC to accelerate hole transfer, the BVO@C achieves an outstanding photocurrent density up to 5.9 mA cm^−2^ at 1.23 V_RHE_ under 1 sun irradiation (AM 1.5 G, 100 mW cm^−2^), and exhibits robust durability within 120 h operation due to carbon shell reinforced corrosion resistance.

## 2 Results and Discussion

**Figure 1:**
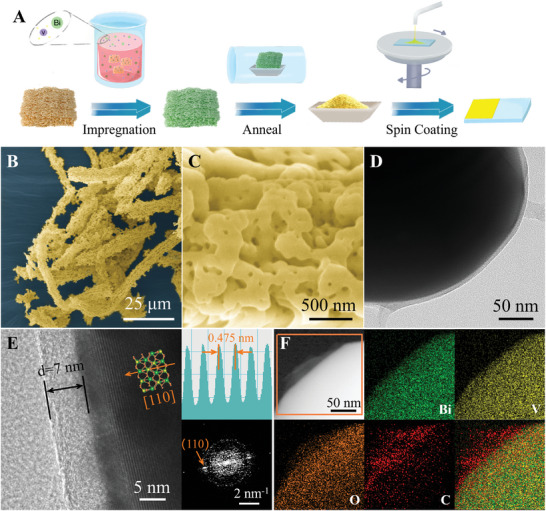
A Schematic synthesis procedure of BVO@C photoanode. B,C) SEM images with different scale bars of BVO@C. D) TEM image and E) HRTEM image of BVO@C with the crystal model, AI, and FFT patterns corresponding to BVO lattice. F) EDS elemental maps of BVO@C.

**Figure** **1**A shows the synthetic procedure used to fabricate BVO@C electrode. The pretreated porous loofah was soaked in the Bi(NO_3_)_3_/NH_4_VO_3_ mixed solution as a biological template to adsorb Bi and V ionic sources. After sufficient impregnation, the loofah changed in color from the original earthy yellow to dark green, and then was annealed in the tube furnace at 600 °C, finally obtaining the bright yellow BVO@C powder sample. Through spin‐coating process, the nanostructured BVO@C catalyst was coated on the fluorine‐doped tin oxide glass (FTO), which could be directly used as the PEC photoanode. The scanning electron microscopy (SEM) image of as‐synthesized BVO@C (Figure 1B) shows the morphology is successfully templated by the porous fibre structure of the loofah. The magnified SEM image (Figure 1C) displays the nanoporous architecture composed of wormlike particles with a size range of 200–300 nm. The transmission electron microscopy (TEM) image (Figure 1D) focusing on a single particle reveals a well‐defined hierarchy with an ultrathin conformal smooth coating. The high‐resolution TEM image (Figure 1E) identifies the inner BVO crystalline and the outer amorphous shell with a uniform thickness of ≈7 nm. The lattice fringe spacing of 0.475 nm corresponds well to the (110) plane of monoclinic BVO phase, and its good crystallinity is further evidenced by the corresponding atomic intensity (AI) profile, and fast Fourier transform (FFT) pattern and selected area electron diffraction (SAED, Figure S1, Supporting Information), respectively. The X‐ray diffraction (XRD) pattern (Figure S2, Supporting Information) also discloses the crystallization of monoclinic BVO (JCPDS No. 00‐014‐0688). The energy dispersive spectroscopy (EDS) elemental maps (Figure 1F) of BVO@C confirms the core‐shell structure of BVO core encased with ultrathin carbon shell, further evidenced by the line sweep EDS spectra across grain edge (Figure S3, Supporting Information). To verify the necessity of biogenic templating to prepare porous BVO@C heterostructure, the blank sample was also synthesized via direct heat anneal without introducing loofah sponge as a vector, which did not exhibit structural homogeneity and carbon encapsulation (Figure S4, Supporting Information).

Note that here a concept of external ETL is proposed mainly to solve the electron transport problem in the thickened optical absorber layer. Hence, we first regulated the thickness of BVO@C film through controllable spin coating on FTO substrates (Figure S5, Supporting Information). As expected, thicker BVO@C layer shows higher optical absorbance (OA) as well as light harvesting efficiency (LHE) (Figure S6, Supporting Information). Accordingly, theoretical photocurrent density (*J*_ABS_) that can be determined by an entire yield of collected photons to conductive electrons increases with increasing layer thickness (Figure S7, Supporting Information). And when the layer thickness reaches ≈811 nm, the *J*_ABS_ reaches saturation, which means a critical thickness just enough for optical absorption. Thus, we selected a BVO@C electrode with 811 nm in thickness as a typical representative to study the charge transfer properties, and also prepared a coating‐free bare BVO electrode with the same thickness by traditional electrodeposition method as a reference.^[^ ^19^ ^]^ The UV–vis absorption spectra of BVO and BVO@C photoanodes show that they have similar optical absorbance and same cut‐off edges at ≈518 nm (Figure S8, Supporting Information), consistent with the bandgap at ≈2.41 eV of BVO. By depicting the wavelength‐dependent absorbance curves within the AM 1.5 G solar spectrum, the *J*_ABS_ values of BVO@C and BVO photoanodes were estimated to be 6.121 and 6.058 mA cm^−2^, respectively (Figure S9, Supporting Information), indicating that they have very similar LHEs.

**Figure 2:**
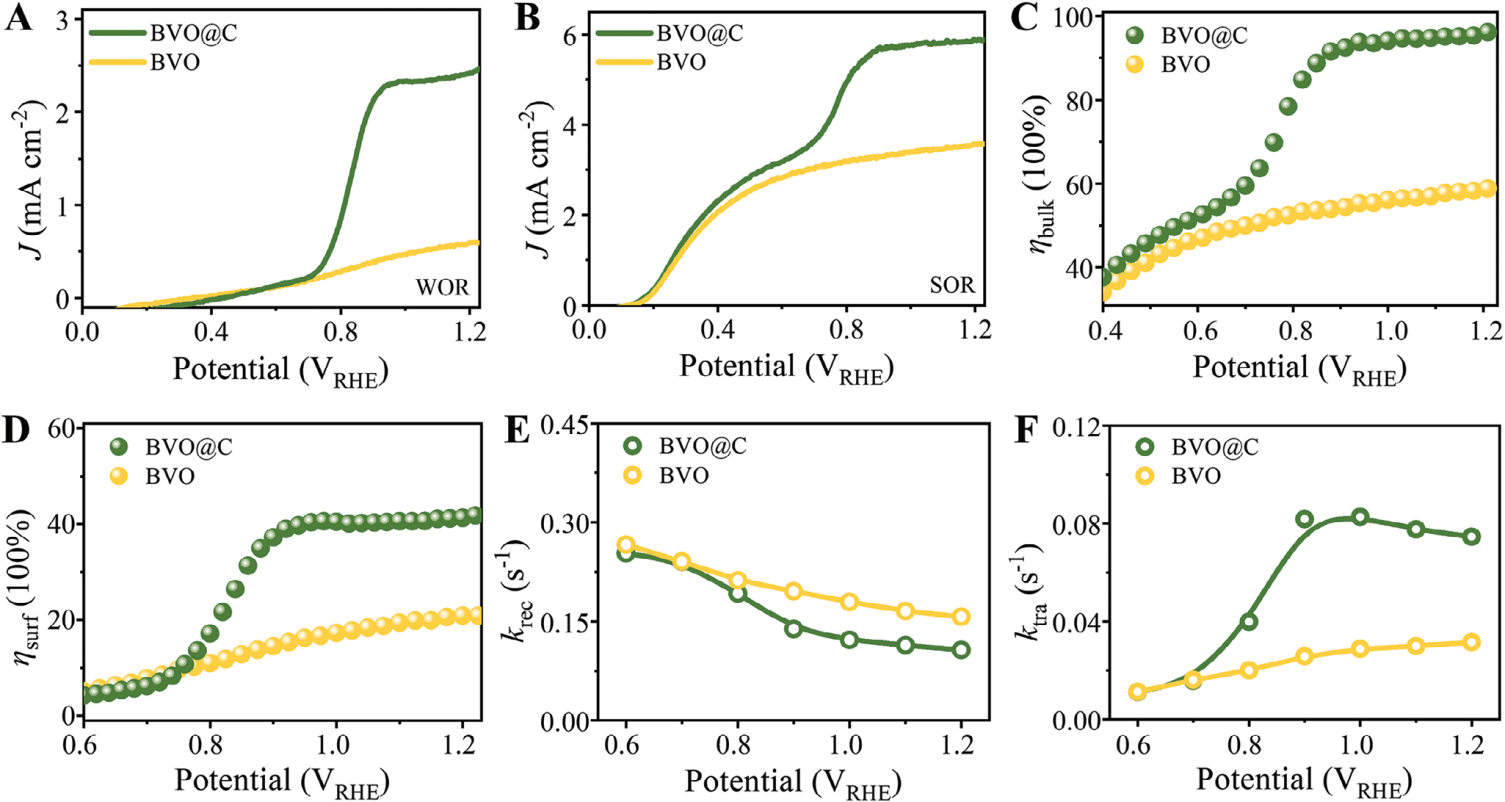
LSV spectra of A) WOR and B) SOR for BVO and BVO@C photoanodes under 1 sun illumination. Charge separation efficiencies C) in bulk and D) at surface for BVO and BVO@C photoanodes. Rate constants of E) charge transfer and F) recombination at surface for BVO and BVO@C photoanodes.

The PEC performance of BVO@C and BVO were measured by linear sweep voltammetry (LSV) at a scan rate of 10 mV s^−1^ in 0.5 m borate buffer solution (pH≈9.3) under front‐side irradiation (AM 1.5 G, 100 mW cm^−2^). As shown in **Figure** **2**A, BVO@C starts the photocurrent density (*J*_WOR_) for water oxidation reaction (WOR) slightly later than BVO with the applied potential increasing, but affords a steep increase when beyond ≈0.7 V_RHE_ and then reaches a high *J*_WOR_ up to 2.47 mA cm^−2^ at 1.23 V_RHE_, which is over four times higher than that for BVO (0.59 mA cm^−2^). Such polarization characteristics indicate an undisputed effect of carbon shell on the *J*_WOR_ growth, but that is independent of intrinsic WOR activity and instead dependent on the external potential field, suggesting a conceivable mechanism associated with enhanced charge separation upon electron transport acceleration. In view of their similar LHEs, the enhancement of *J*_WOR_ observed for BVO@C can be ascribed to the charge separation efficiencies in bulk (η_bulk_) or at surface (η_surf_). To decouple charge dynamics, we further measured the photocurrent density (*J*_SOR_) for sulphite oxidation reaction (SOR) by introducing 0.2 M Na_2_SO_3_ as surface hole scavenger that can eliminate the surface charge recombination (*R*_surf_) (Figure 2B). In this case, the η_bulk_ and η_surf_ can be calculated by decoupling method based on the Equations S3 and S4 (Supporting Information). Figure 2C shows that an impressive η_bulk_ value up to 96% is achieved for BVO@C, much superior to BVO (59%), indicating the enhanced bulk charge transport in BVO@C. In particular, a sharp increase in η_bulk_ after 0.7 V_RHE_ implies that there is different transport pathway for charge separation acceleration instead of BVO itself. Based on PEC comparison for sulphite oxidation under front‐side and back‐side irradiation (Figure S10, Supporting Information), the transport dynamics of majority electrons and minority holes in bulk were further analyzed in Figure S11 (Supporting Information). For pristine BVO, the η_bulk_ is higher under back‐side irradiation than that under front‐side irradiation, indicative of sluggish electron transport property in BVO, which is in line with previous findings on heavy electron transport barriers in porous BVO layer thickened over 200 nm.^[^ ^17^, ^39^ ^]^ In contrast, higher η_bulk_ is observed under front‐side illumination rather than back‐side illumination for BVO@C, which indicates that the bulk electron transport is substantially improved via the outer carbon pathway around BVO. Hence, the enhanced η_bulk_ can be attributed to the fact that the accelerated electron transport by external conductive channels under applied potentials effectively reduces the bulk charge recombination (*R*_bulk_).

Figure 2D shows that the η_surf_ values for both BVO@C and BVO are relatively low (<50%), and this is reasonable because there is no cocatalyst loaded on the surface to accelerate the hole transfer toward WOR. Nevertheless, BVO@C still exhibits higher η_surf_ in comparison with BVO, especially after the external potential exceeds 0.7 V_RHE_. We further extracted the dynamic rate constants of charge recombination (*k*_rec_) and transfer (*k*_tra_) on surface, according to the photocurrent decay from spike to steady states along with *R*_surf_ upon illumination onset (Figure S12, Supporting Information). The lower *k*_rec_ in BVO@C relative to BVO suggests that the carbon coating may play an additional role in restraining *R*_surf_ that is commonly serious especially on the defective surface with charge‐trapping sites (Figure 2E). On the other hand, the *k*_tra_ in BVO@C increases with external potential and overtakes that in BVO when beyond 0.7 V_RHE_ (Figure 2F), which indicates that the improved surface hole transfer in BVO@C originates from higher charge separation by suppressing both bulk and surface recombination, rather than the co‐catalytic effect of outer carbon shell. Besides, the almost unresponsive LSV curves for water oxidation under dark field of BVO@C similar to BVO (Figure S13, Supporting Information) also deny the contribution of carbon shell to electrocatalytic WOR activity. So, higher η_surf_ in BVO@C can be attributed to the reduced surface recombination by charge‐trapping defect passivation and the enhanced surface transfer by hole accumulation with long survival. In addition, the photocurrent density curves upon illumination switch and EPR‐electron capture curves further confirm the higher photoelectron yield against charge recombination for BVO@C compared with BVO (Figure S14, Supporting Information). Further, the no‐voltage time‐resolved transient absorption spectroscopy (TAS) (Figure S15, Supporting Information) describes the carriers’ dynamics in the biexponential decay with two fitting components *τ*_1_ and *τ*_2_ ascribed to the shallow trapping and the charge recombination, respectively.^[^ ^40^ ^]^ Thus, the increased *τ*_1_ and *τ*_2_ in BVO@C with respect to pristine BVO reveal the relatively few electrons trapped by the shallow defects for BVO@C. Together, the carbon coating accelerates charge transport and separation, responsible for the increased *J*_WOR_ in BVO@C photoanode.

**Figure 3:**
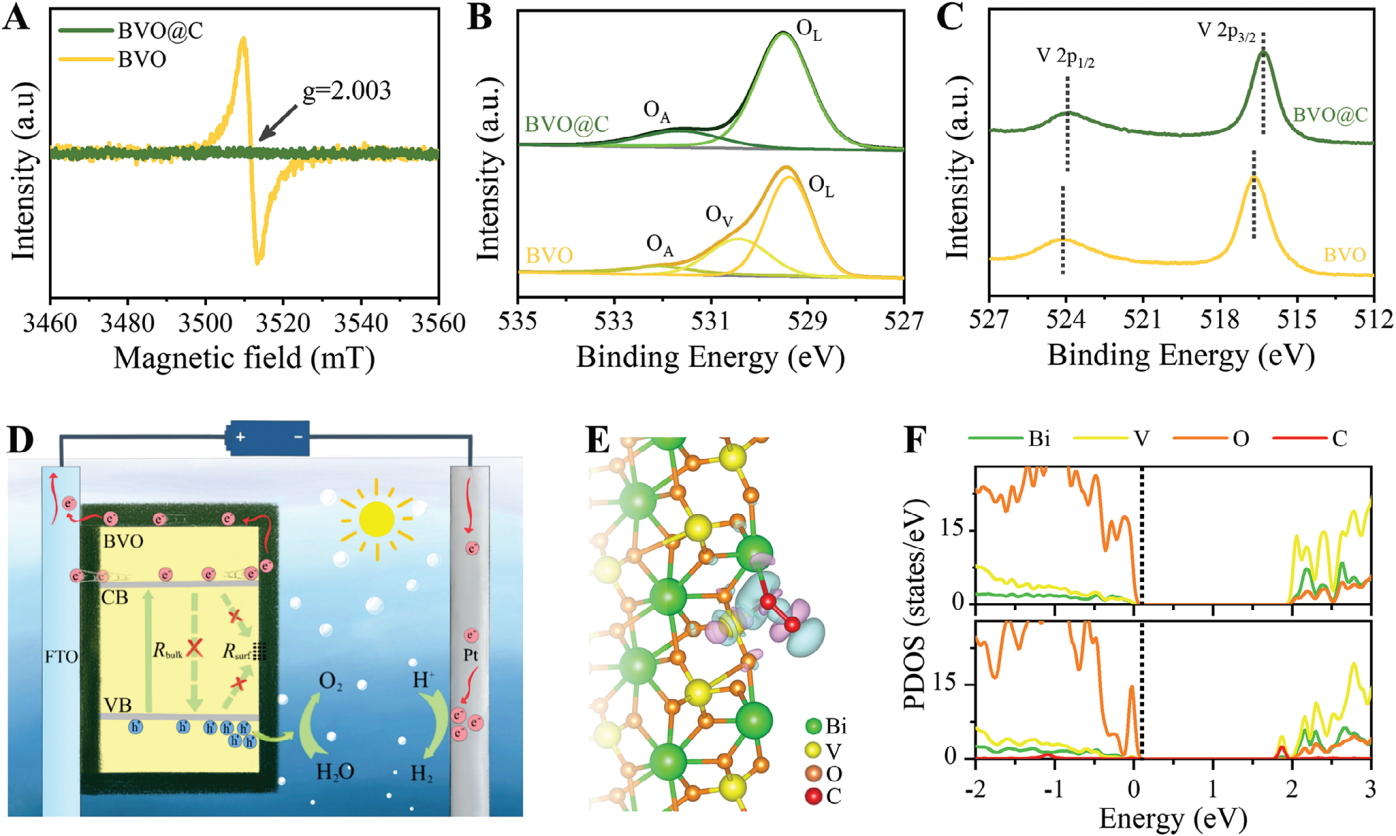
A) EPR spectra, and B,C) high‐resolution XPS spectra of O 1s and V 2p for BVO@C and BVO photoanodes. D) Schematic diagram of charge transport dynamics in BVO@C photoanode. E) DFT structure with Bader charge distribution around carbon‐coordinated bonds. Note: the electron accumulation in blue zone and depletion in purple zone. F) Calculated PDOS patterns for BVO@C and BVO.

To gain deeper insight into the special role of the carbon coating in boosting charge transport and separation, the electron paramagnetic resonance (EPR) spectroscopy and X‐ray photoelectron spectroscopy (XPS) were performed to analyze surface chemical states. **Figure** **3**A shows a noticeable EPR signal of oxygen vacancies (V_O_) for bare BVO, which locate mostly on the surface because of the perfect crystallinity inside. Interestingly, BVO@C does not display characteristic EPR signal, ascertaining an effective passivation of surface defects by carbon coating to suppress *R*_surf_. High‐resolution XPS spectra identify the changes in V 2p‐core and O 1s‐core peaks for BVO@C compared to BVO, while the signal peaks of other atoms remain unchanged (Figure S16, Supporting Information). The increase in signal peak at ≈288 nm in C 1s XPS spectra indicates the carbon decoration in C@BVO. As shown in Figure 3B, the O 1s spectrum of BVO can be deconvolved into three peaks centered at 529.4, 530.4, and 532.1 eV, which are assigned to oxygen ions in the lattice (O_L_), hydroxy radical bonded to cations around V_O_ defects (O_V_), and adsorbed oxygen‐containing species (O_A_), respectively.^[^ ^41^ ^]^ The O_V_ peak quenching in O 1s spectrum of BVO@C further identifies the effect of carbon coating to passivate surface defects. In Figure 3C, two spin‐orbit peaks of V 2p appear an obvious redshift for BVO@C with respect to BVO,^[^ ^42^ ^]^ which reveals strong electronic modulation by C‐V coordination. Note that the photogenerated electron and hole polarons in BVO crystal are localized at VO_4_ tetrahedron and BiO_8_ octahedron unites,^[^ ^43^ ^]^ respectively. The previous studies have pointed out that the charge carriers bound in polaron states would be transported in hopping model rather than conducting model in the lattice,^[^ ^44^, ^45^ ^]^ severely impeding bulk charge migration. Here, surface V polarly coordinated with C would induce electrons in conduction band (CB) to migrate from VO_4_ unites into outer C atoms, which is suggested to be efficient for separating electron‐hole polarons. Moreover, because the carbon envelope forms a 3D connected network to receive the direct action of the external electrical potential without interference from low conductive BVO layer, the electrons can be transported smoothly to the FTO substrate through high conductive channels established by C─C bonds in the carbon layer. Therefore, it can be concluded that the carbon overlayer serves as both an antenna for collecting electrons and a channel for transporting electrons, avoiding the sluggish mobility of charge polarons within BVO lattice and the formidable barrier at grain junctions, which thus successfully alleviates both the *R*_bulk_ and *R*_surf_. In view of this mechanism shown schematically in Figure 3D, we refer the carbon coating to as an external ETL, which differs from both the traditional ETL that usually is designed underneath the absorber layer. Carbon materials have widely used to capture, store, and shuttle electrons, especially in supercapacitors.^[^ ^46^ ^]^ Wang et al. ever employed carbon spheres as the electron reservoirs to capture the photogenerated electrons of BVO particles via random contacts in their particle‐mixed film.^[^ ^47^ ^]^ In contrast, an intimate carbon conjunction induced by biological templates here is more competent to collect and conduct electrons, and thus appears more effective for improving charge separation.

The band bending upward at the interface of an n‐type semiconductor photoanode (i.e., an outward electric field built in the charge depletion region) is conducive to the hole transfer outward,^[^ ^48^, ^49^ ^]^ so the traditional ETL needs to be buried underneath the semiconductor layer rather than facing the electrolyte on the outside.^[^ ^8^ ^]^ However, here the inverted band bending by an external ETL allows the electrons to be exported and sent away preferentially, leaving the holes to survive longer and accumulate at the VB with enhanced oxidative potential favourable for WOR. This will induce a dynamic band structure with interfacial double‐layer capacitors, similar to the switched charge–discharge dynamics, which was also defined as the bipolarized Faraday junction by Luo et al.^[^ ^50^ ^]^ Therefore, the outer carbon layer acts no direct modulation on hole intrinsic dynamics, and instead loses the propensity of spontaneous hole transfer at low potential. This is reason why the above dynamic efficiencies and rates increase only after 0.7 V_RHE_, beyond that the electron transport acceleration via an external ETL boosts charge separation and hole survival in the valence band (VB) along with oxidative potential growth beneficial to WOR.

We further employed density functional theory (DFT) calculations to understand the passivation effect of carbon atoms on oxygen defective surface with two contrast structures (Figure S17, Supporting Information). Figure 3E shows the Bader charge distribution around C‐coordinated bonds on the passivated surface, revealing the electron accumulation at C atom and depletion at V atom (Figure 3E), and such charge‐polarized bonds would mediate the rapid transfer of photogenerated electrons located in VO_4_ unit to C atom. Interestingly, with the C atom introduced, emerging C orbital at the CB bottom in the projected density of states (PDOS) is hybridized with V orbital (Figure 3F), which can account for the function of C‐coordination in extracting electrons from V 3d CB for suppressing bulk recombination. Such electron transfer breaks the polaron shackles within BVO, by that more effective electron transport via an external ETL is responsible for enhanced charge separation for BVO@C.

**Figure 4:**
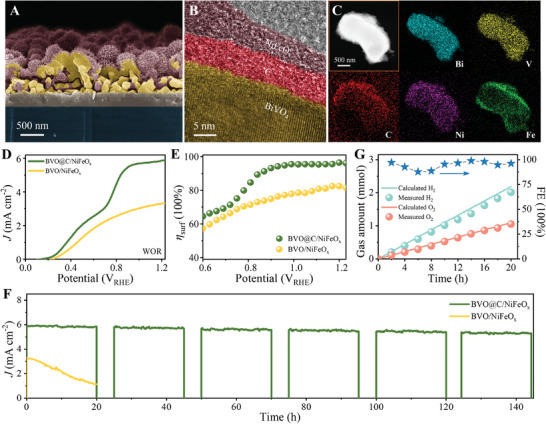
A) SEM image, B) TEM image and C) EDS elemental mappings of BVO@C/NiFeO_x_. D) LSV spectra for water splitting under 1 sun illumination, and E) calculated η_surf_ for BVO@C/NiFeO_x_ and BVO/NiFeO_x_ photoanodes. F) Long‐term durability testing with a few 5 h blackout intervals for BVO@C/NiFeO_x_ and BVO/NiFeO_x_ photoanodes. G) Measured and calculated amounts of gas products over BVO@C/NiFeO_x_ based PEC cell for the FE assessment.

For further enhancing the PEC performance of water oxidation, BVO@C photoanode was decorated with nanosized nickel‐iron oxide (NiFeO_x_) as an OEC on the surface to improve the η_surf_. The SEM image shows that a thin layer of flocculent NiFeO_x_ is uniformly covered on BVO@C surface (**Figure** **4**A), which notarizes the successful decoration of an OEC layer. The TEM image displays a clear hierarchical sandwich structure (Figure 4B), further indicating the uniform loading of amorphous NiFeO_x_ layer on BVO@C particles with an average thickness of ≈5 nm. The EDS mappings visualize the homogeneous locations of Bi, V, C, Ni and Fe elements within a single BVO@C/NiFeO_x_ particle (Figure 4C); moreover, the distributions of Ni and Fe elements are slightly wider than those of Bi and V elements under close contrast observation. In addition, the XRD pattern and UV–vis spectra of BVO@C/NiFeO_x_ (Figure S18, Supporting Information) manifest that the loading of NiFeO_x_ induces negligible influences on the crystal structure and optical absorption of BVO@C. As expected, the faster *J*_WOR_ growth accompanied by the cathodic shifts of onset potentials were observed for both BVO and BVO@C after the decoration of NiFeO_x_ cocatalyt in the water‐oxidation LSV curves (Figure S19, Supporting Information), verifying the enhanced WOR kinetics by NiFeO_x_ cocatalyst. Impressively, the *J*_WOR_ of BVO@C/NiFeO_x_ is increased to ≈5.9 mA cm^−2^ at 1.23 V_RHE_ (Figure 4D), which is among the front rank for single‐absorber BVO photoanodes (Table S1, Supporting Information). To the best of our knowledge, the record photocurrent density for single‐absorber BVO photoanode was updated recently by Bi's group with different novel cocatalysts, such as 6.4 mA cm^−2^ at 1.23 V_RHE_ with N‐doped NiFeO_x_ cocatalyst in 2021^[^ ^24^ ^]^ and 6.73 mA cm^−2^ at 1.23 V_RHE_ with NiFePO_x_ cocatalyst in 2022.^[^ ^47^ ^]^ Note that in their reports the conventional NiFeO_x_ cocatalyst for comparison raises the photocurrent density to only 4.4 mA cm^−2^ at 1.23 V_RHE_. In comparison, our BVO@C has advantages in charge transport and separation, while leaving room of cocatalyst optimization to improve the PEC water splitting performance. In addition, BVO@C/NiFeO_x_ photoanode achieves a η_surf_ up to ≈96.4% at 1.23 V_RHE_ (Figure 4E) and an applied bias photon‐to‐current efficiency (ABPE) maximum of ≈1.96% at 0.83 V_RHE_ (Figure S20, Supporting Information), both of which are higher than those of BVO/NiFeO_x_ photoanode. The electrochemical impedance spectroscopy (EIS) measurements reveal the smallest semicircular diameter in Nyquist plots for BVO@C/NiFeO_x_, indicating more efficient charge transfer compared to BVO and BVO@C due to the NiFeO_x_ cocatalyst (Figure S21, Supporting Information). Further, the incident photon‐to‐current conversion efficiency (IPCE) curves were measured for BVO, BVO@C, and BVO@C/NiFeO_x_ photoanodes at 0.83 V_RHE_ with specific single‐wavelength filters (Figure S22, Supporting Information). The three photoanodes show similar photocurrent responsive range of 350–510 nm, which corresponds to the absorption spectra with cut‐off edges (Figure S8, Supporting Information), indicating that the photocurrents intrinsically derive from the bandgap transition of BVO upon light excitation for all three photoanodes. The maximum IPCE values obtained with BVO@C/NiFeO_x_ verify the contributions from ETL and cocatalyst layers to charge transport and separation.

The PEC durability was investigated at 1.23 V_RHE_ by long‐term chronoamperometry (CA) course with a few 5 h blackout intervals. As shown in Figure 4F, BVO@C/NiFeO_x_ exhibits the steady photocurrent density within 120 h switch‐on operation, indicating an excellent stability for PEC water splitting. Whereas, BVO/NiFeO_x_ undergoes an obvious decay of photocurrent density in the first 20 h CA operation at same 1.23 V_RHE_. In situ inductively coupled plasma mass spectrometry (ICP‐MS) measurements (Figure S23, Supporting Information) discover the deactivation cause associated with the V ion dissolution, which can be effectively inhibited by the carbon shell protection. Though further post‐characterizations for BVO@C/NiFeO_x_ electrode, the original morphology, hierarchical structure, and heterogeneous composition keep almost unchanged after the long‐term durability test (Figure S24, Supporting Information). This suggests that the carbon encapsulation not only mediates the charge dynamics but also enables the resistance to corrosion. Note that PEC stability tests of BVO photoanode were performed commonly for no >50 h in laboratory (Table S1, Supporting Information),^[^ ^48^, ^51^ ^]^ and thus here the steady delivery of photocurrent density as high as 5.9 mA cm^−2^ at 1.23 V_RHE_ over 120 h represents the best record of activity‐stability tradeoff. The gas evolution amounts were measured by gas chromatography (GC) (Figure S25, Supporting Information), where O_2_ and H_2_ evolving accords nearly with the theoretical curves calculated by photocurrent density and corresponds well to the stoichiometric 2:1 ratio of overall water splitting (Figure 4G). The calculated Faraday efficiency (FE) is close to 100%, indicating that the overall water splitting proceeds without other side reactions or by‐products.

## 3 Conclusion 

In summary, we demonstrate the feasibility of an external ETL to improve charge separation by using the carbon envelope on BVO particles as an interconnect network for electron extraction and transport. This design allows the thickening of absorber layer to maximize the light harvesting, as well as meanwhile eliminates the charge‐trapping defect sites on the surface and separates the semiconductor from the electrolyte to enhance corrosion resistance. As a result, BVO@C/NiFeO_x_ photoanode achieves an impressive photocurrent density up to 5.9 mA cm^2^ at 1.23 V_RHE_ under 1 sun illumination, along with an excellent durability over 120 h, which are among the best records for single BVO absorber photoanodes. Such an external ETL concept opens up a novel avenue to address the poor bulk charge transport that embarrasses most oxide semiconductors for designing high‐performance PEC energy systems.

## Conflict of Interest

The authors declare no conflict of interest.

## Author contributions

T.Y., J.C. and L.L. contributed equally to this work.X.X. conceived the research. X.X. and X.Y. designed the experimental setup. X.Y., L.L., J.C., and W.D. carried out the material synthesis, structural characterizations and photoelectrochemical measurements. A.B. and J.D. performed TAS measurements and analysis. S.G. and J.H. performed theoretical calculations and data analysis. X.X. and X.Y. wrote the manuscript. All authors engaged in the result discussion and manuscript edition.

## Supporting information

Supporting Information

## Data Availability

The data that support the findings of this study are available from the corresponding author upon reasonable request.
