# Supplementary material for: Enhanced Charge Separation in Nanoporous BiVO4 by External Electron Transport Layer Boosts Solar Water Splitting
Source: Adv Sci (Weinh). 2023 Dec 7;11(5):2305567. doi: 10.1002/advs.202305567 (PMC10837342; doi:10.1002/advs.202305567)
Supplement: Supplementary file 1 — Supporting Information [file ADVS-11-2305567-s001.pdf]

ADVANCED SCIENCE
Open Access

Supporting Information

for *Adv. Sci.*, DOI 10.1002/advs.202305567

Enhanced Charge Separation in Nanoporous BiVO4 by External Electron Transport Layer Boosts Solar Water Splitting

Xiaotian Yang, Jianpeng Cui, Luxue Lin, Ang Bian, Jun Dai, Wei Du, Shiying Guo, Jingguo Hu and Xiaoyong Xu**

Supporting Information

Enhanced Charge Separation in Nanoporous $BiVO_4$ by External Electron Transport Layer Boosts Solar Water Splitting

Xiaotian Yang, Jianpeng Cui, Luxue Lin, Ang Bian, Jun Dai, Wei Du, Shiying Guo, Jingguo Hu, and Xiaoyong Xu**

Dr. X. Yang, Dr. J. Cui, Miss L. Lin, Dr. W. Du, Dr. S. Guo, Prof. J. Hu, and Prof. X. Xu

College of Physics Science and Technology, and Interdisciplinary Research Center, Yangzhou University, Yangzhou 225002, China.

Dr. A. Bian, Prof. J. Dai

School of Science, Jiangsu University of Science and Technology, Zhenjiang 212100, China.

*Corresponding Author.

E-mails: xxy@yzu.edu.cn (X.X.) and jghu@yzu.edu.cn (J.H.)

Keywords: photochemical cell, $BiVO_4$ photoande, solar water splitting, charge transport

1. Experimental Section

Materials: All chemicals were used as received without further purification. 25% glutaraldehyde, 37% hydrochloric acid, 65% nitric acid, Tetramethylammonium hydroxide (TMAH), NH_4VO_3, $Bi(NO_3)_3·5H_2O$, ethanol and glycerol were purchased from Guangning Co., Ltd., China. The loofah comes from the College of Agriculture of Yangzhou University.

Synthesis of BVO@C sample: BVO@C was fabricated by using loofah as a template followed by impregnation and calcination processes. Prior to the synthesis, the loofah was pretreated by sequentially soaking it in 2% glutaraldehyde phosphate buffer for 8 h and in 5% hydrochloric acid for 3 h, and then rinsed with deionized water (DIW). Adding 40 mmol Bi $(NO_3)_3·5H_2O$ into a mixture of 80 mL ethanol and 60 mL glycerol obtains a solution of Bi source. Adding 40 mmol NH_4VO_3 into 40 mL of TMAH obtains a solution of V source. The two solutions were mixed with stirring at 70 ℃, appearing large amounts of yellow precipitate, and then nitric acid (65%) was added dropwise timely until the solution became transparent, forming the impregnation solution. The treated loofah was immersed in above solution for 10 h for impregnating with Bi and V sources, and then rinsed fully with ethanol/DIW. Finally, the impregnated loofah was calcined in a tubular furnace for 6 h at 600 ℃ in air, obtaining the nanoporous BVO@C sample. In order to demonstrate the function of the porous loofah template, we also directly calcined the impregnation without introducing the loofah, and the obtained sample no longer had porous structure and carbon coating. The BVO@C electrodes were fabricated by spin-coating BVO@C stock on the size-normalized fluorine-doped tin oxide (FTO) glasses (FTO: 1 cm × 2 cm, sample area: 1 cm × 1 cm), with BVO@C layer thickness regulation. For

comparison, the nanoporous bare BVO electrode was also prepared through the previously reported process of electrodeposition followed by thermalization.

Modification with $NiFeO_x$ cocatalyst: $NiFeO_x$ deposition was carried out using a three-electrode PEC cell in 0.5 M potassium borate electrolyte containing 20 mM $Ni(NO_3)_2{\cdot}6H_2O$ and 20 mM $Fe(NO_3)_2{\cdot}6H_2O$. Ag/AgCl electrode and Pt sheet were employed as reference and counter electrodes, respectively. Through the cyclic voltammetry (CV) scanning at a rate of 50 mV s^{-1} within -0.4-0.4 V vs. Ag/AgCl under AM 1.5 G illumination (from front side) for two cycles, $NiFeO_x$ cocatalyst was deposited onto BVO and BVO@C electrodes.

Characterization: SEM was taken on a S-4800 microscope (Hitachi, Japan). TEM and EDS were performed on a G2 F30 S-TWIN microscope (Tecnai, America) coupled with an EDS module at 300 kV. XRD patterns were recorded on a D8 Advance diffractometer (Bruker, Germany) equipped with a Cu-Kα radiation source. XPS spectra were obtained on an ESCALAB250Xi instrument (ThermoFisher, America) equipped with an Al-Kα source. EPR spectroscopy was carried out on an A300-10/12 spectrometer (Bruker, Germany). UV-Vis spectra were measured with a Cary 5000 spectrophotometer (Varian, America). The TAS spectra were recorded with a self-made system (TIME-TECH SPECTRA) under the pump excitation of 343 nm.

PEC Measurements: PEC performance was measured with a typical three-electrode system (CHI660d, Shanghai CH) using an Ag/AgCl reference electrode and Pt foil counter electrode under a Xe 300 W lamp (Microsolar 300, Beijing Perfectlight) as the light source coupled with an AM 1.5 G filter. The light intensity was uniformly calibrated to 100 mW cm^{-2} (1 sun) by an optical power meter (FZ-A, Beijing

Perfectlight). 0.5 M potassium borate buffer solutions (pH 9.3) with and without 0.2 M Na_2SO_3 as a sacrificial agent were used as the electrolytes for SOR and WOR measurements, respectively. LSV curves were recorded at a scan rate of 50 mV s^{-1} within a potential range of 0-1.3 V_{RHE} to evaluate photocurrent densities. The stability tests in chronoamperometry were conducted at 1.23 V_{RHE} for 145 h with several light-off phases of 5 h. IPCE curves were measured at 0.83 V_{RHE} with specific single-wavelength filters. The gas-evolving amount was measured with a gas-tight system coupled with PEC cell (Labsolar-IIIAG, Beijing Perfectlight) and gas chromatograph (GC, Fuli, Zhejiang).

Theoretical formulas: All the potentials were converted versus RHE according to the following Nernst equation

$$E_{\mathrm{RHE}} = E_{\mathrm{Ag/AgCl}} + 0.059 \times \mathrm{pH} + E^{0}_{\mathrm{Ag/AgCl}} \quad \text{(S1)}$$

where E_{RHE} and $E_{\mathrm{Ag/AgCl}}$ are the potentials versus RHE and Ag/AgCl, respectively, and $E^{0}_{\mathrm{Ag/AgCl}}$ is 0.1976 V at room temperature (25 °C).

The LHE can be calculated by the following equation

$$\mathrm{LHE} = 1 - 10^{-A(\lambda)} \quad \text{(S2)}$$

where $A(\lambda)$ is the absorbance at specific light wavelength (λ). And the J_{ABS} can be calculated by integrating the LHE spectra with standard spectrum of AM 1.5 G based on an assumption of 100% photon-to-current conversion.

The η_{bulk} and η_{surf} can be calculated by the following equations

$$\eta_{\mathrm{bulk}} = \frac{J_{\mathrm{SOR}}}{J_{\mathrm{ABS}}} \quad \text{(S3)}$$

$$\eta_{\text{surf}} = \frac{J_{\text{WOR}}}{J_{\text{SOR}}} \tag{S4}$$

where J_{SOR} and J_{WOR} are the photocurrent densities of PEC sulfate oxidation and water oxidation, respectively.

The k_{rec} and k_{tra} can be calculated according to the photocurrent transient response spectra by the following equations

$$\frac{J_t - J_\infty}{J_0 - J_\infty} = \mathrm{e}^{-t/\tau} \tag{S5}$$

$$\frac{J_\infty}{J_0} = \frac{k_{\text{tra}}}{k_{\text{rec}} + k_{\text{tra}}} \tag{S6}$$

$$\tau = \frac{1}{k_{\text{rec}} + k_{\text{tra}}} \tag{S7}$$

where J_0 and J_∞ are the instantaneous photocurrent density and the steady-state photocurrent density, and τ is the time constant, respectively.

The ABPE can be estimated by the following equation

$$\text{ABPE (\%)} = \frac{J \times (1.23 - V_{\text{RHE}})}{P_{\text{sun}}} \times 100\% \tag{S8}$$

where J is the photocurrent density at the applied potential (V_{RHE}) versus RHE, and P_{sun} is 1 sun irradiation intensity (100 mW cm^{-2}, AM 1.5 G).

The theoretical amounts of H_2 and O_2 evolution (N_{H}, N_{O}), and the corresponding faradaic efficiency (FE) were calculated by the following equations

$$N_{\text{H}} = \int_0^t \frac{J \times A}{2 \times N_{\text{A}} \times e} \times dt \tag{S9}$$

$$N_{\text{O}} = \int_0^t \frac{J \times A}{4 \times N_{\text{A}} \times e} \times dt \tag{S10}$$

$$\text{FE} = \frac{n_{\text{O}}}{N_{\text{O}}} = \frac{n_{\text{H}}}{N_{\text{H}}} \tag{S11}$$

n_H and n_O are the measured mole amounts of H_2 and O_2 evolutions, respectively. J and A are the recorded current density and the effective area of photoanode, N_A and e are the Avogadro number and the electron charge, t is the gas-collecting time, and the coefficients of 2 and 4 refers to electron numbers involved into each OER and HER.

DFT calculations: All the DFT calculations were performed under periodic boundary conditions using the Vienna ab simulation package (VASP). The projector-augmented-wave (PAW) method with a plane-wave basis set was used. Geometry optimization and electronic properties were carried out under generalized gradient approximation (GGA) with the Perdew, Burke, and Ernzerholf (PBE) exchange-correlation functional. The convergences of energy and force were 1×10^{-4} eV and 0.01 eVÅ^{-1}. A kinetic energy cutoff of 400 eV was set on a grid of 5×5×5 k-point mesh for BVO bulk and 5×5×1 k-point mesh for BVO surface. To properly describe the strongly correlated electrons in the partially filled d subshells, we used the Hubbard Ueff to correct the electronic properties introduced by Dudarev et al. The Hubbard Ueff is defined as Ueff=U−J, where U and J specify the on-site Coulomb and exchange interaction parameters, respectively. The Ueff=2.70 eV for V is extracted from reference 10.1038/ncomms9769.

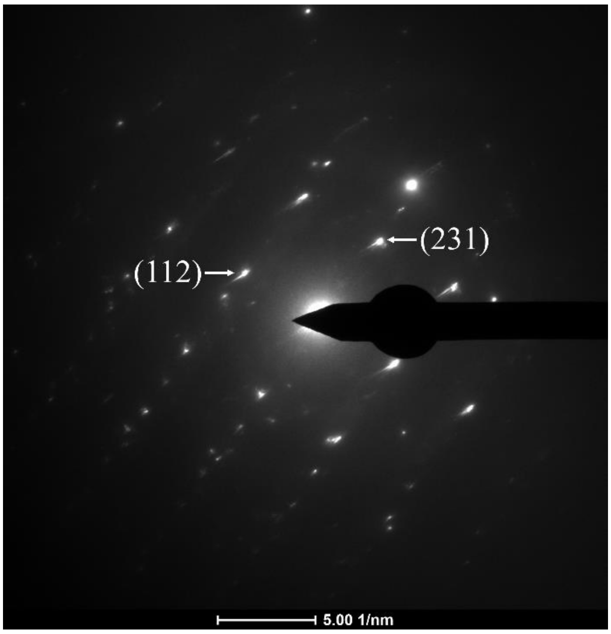

Figure S1. SAED pattern of BVO@C.

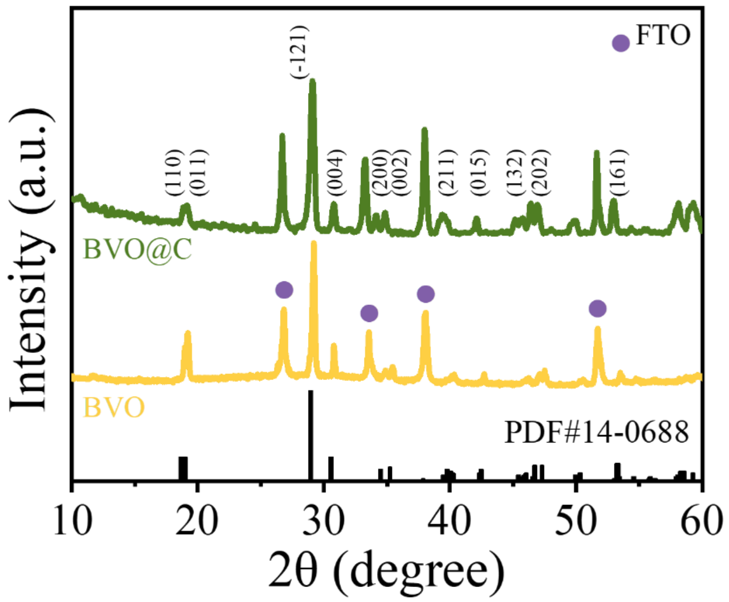

Figure S2. XRD pattern of BVO@C, with BVO as a reference.

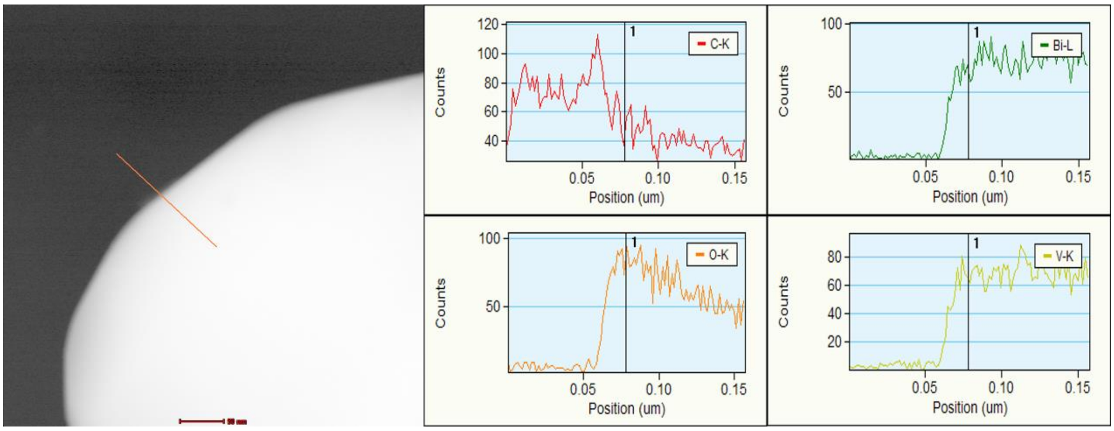

Figure S3. Line sweep EDS spectra across grain edge for C, O, Bi and V elements.

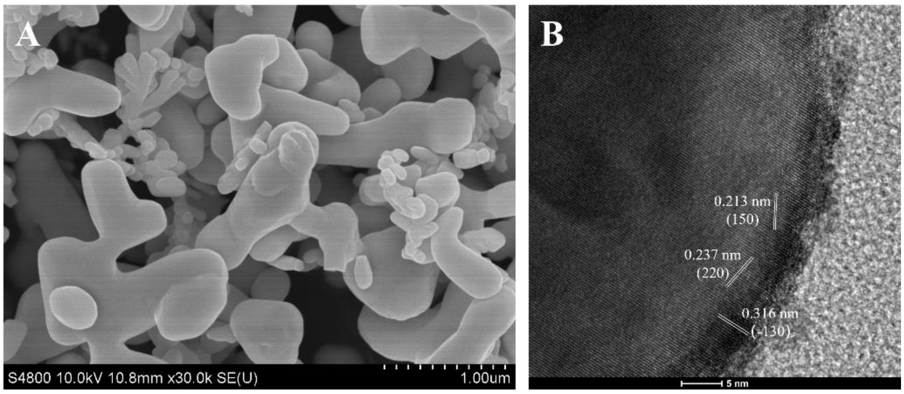

Figure S4. (A) SEM image and (B) TEM image of the blank sample synthesized via direct heat anneal without introducing loofah sponge as a vector, which does not exhibit porosity and carbon encapsulation.

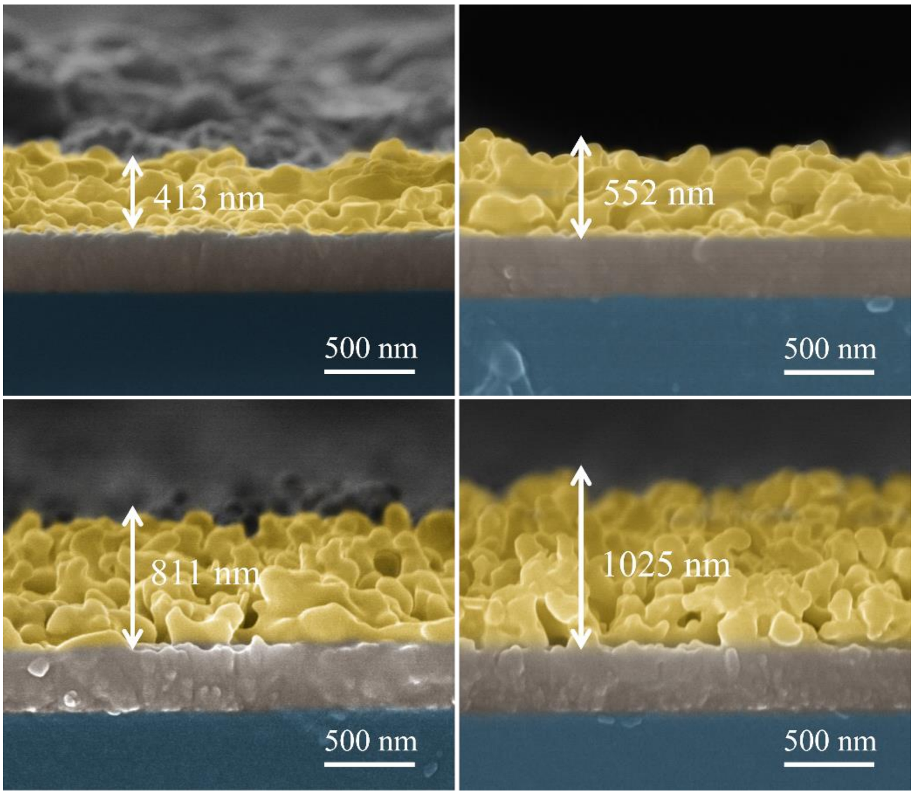

Figure S5. SEM images of BVO@C films coated on FTO with thickness regulation.

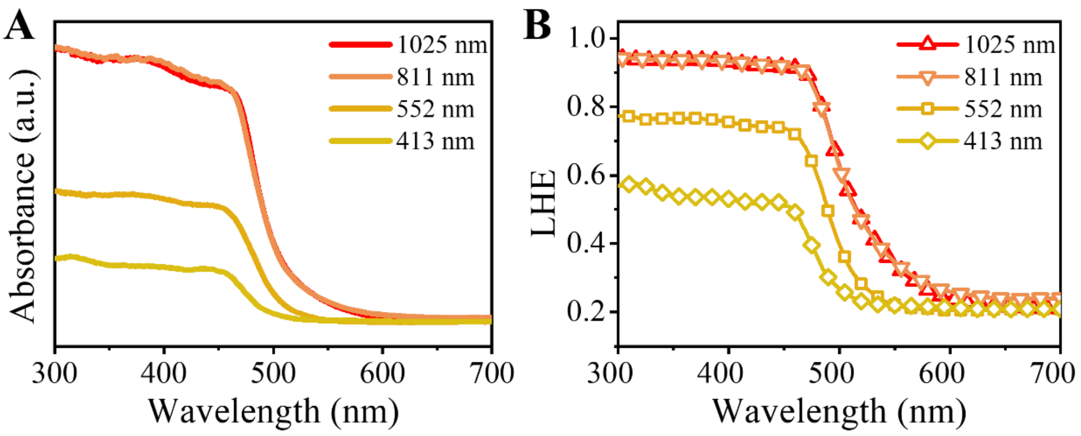

Figure S6. (A) UV-vis absorbance spectra and (B) the corresponding LHE of BVO@C with different thicknesses.

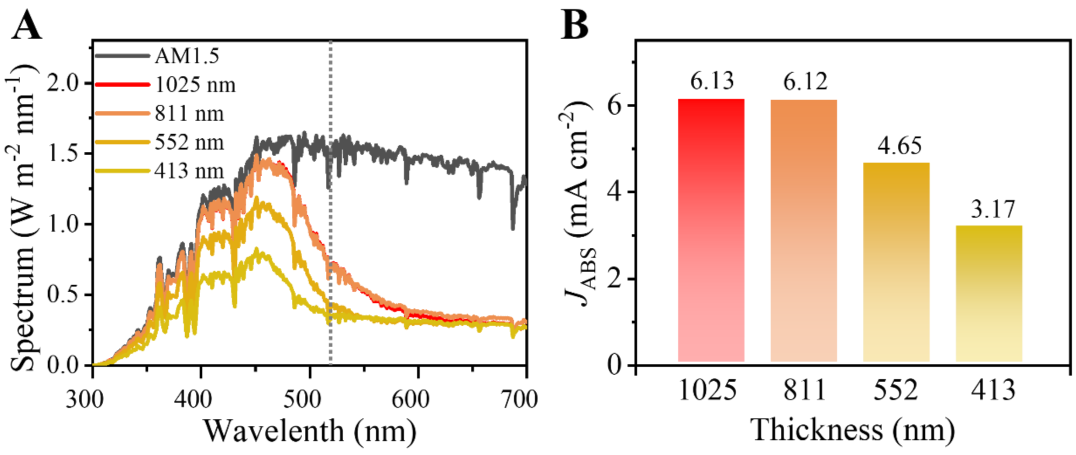

Figure S7. (A) Integrated light absorption spectra within the standard solar of AM 1.5 G and (B) the corresponding J_{ABS} values of BVO@C with different thicknesses.

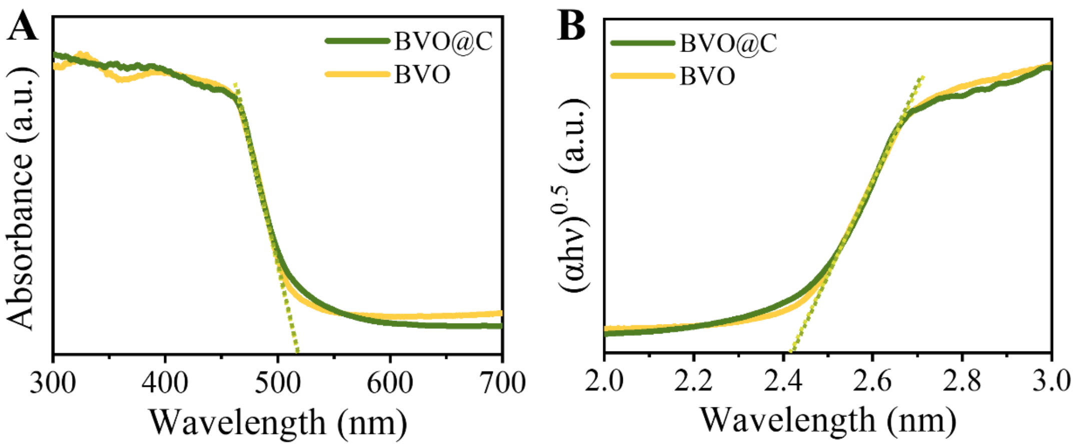

Figure S8. (A) UV-vis absorbance spectra and (B) the corresponding Tauc plots of BVO and BVO@C photoanodes.

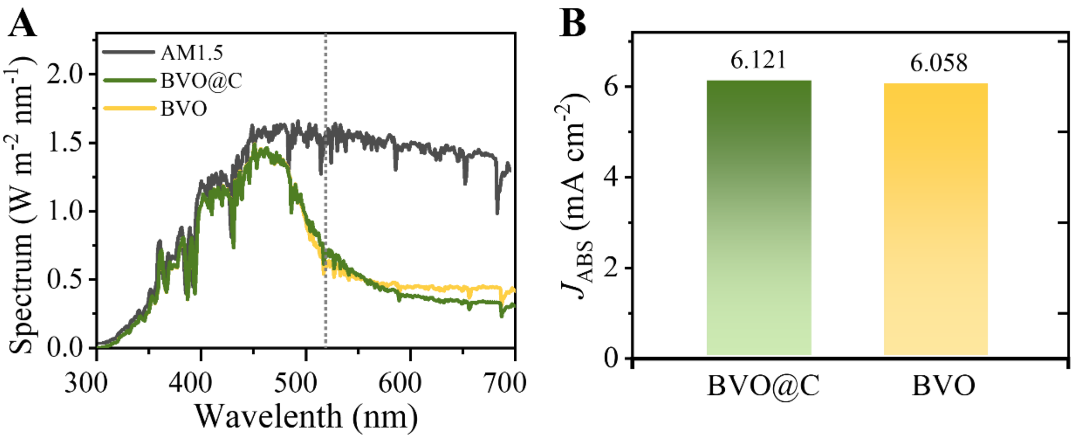

Figure S9. (A) Integrated light absorption spectra within the standard solar of AM 1.5 G, and (B) the corresponding J_{ABS} values of BVO@C and BVO photoanodes.

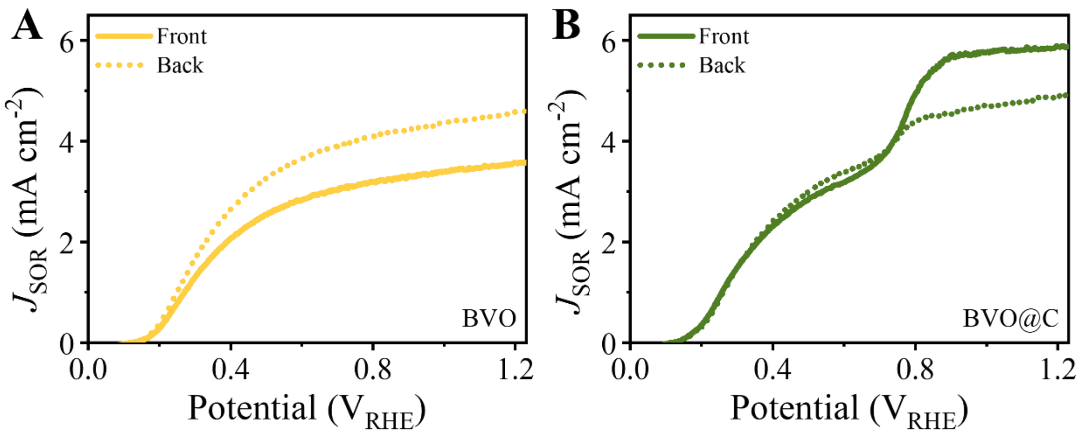

Figure S10. LSV spectra of SOR under front-side and back-side illumination for (A) BVO and (B) BVO@C photoanodes.

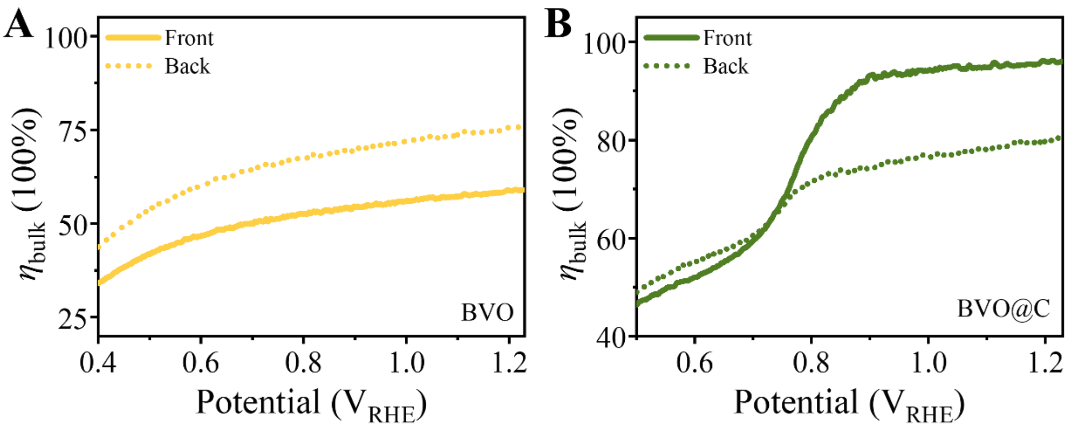

Figure S11. Comparison of η_{bulk} under front-side and back-side illumination in (A) BVO and (B) BVO@C photoanodes.

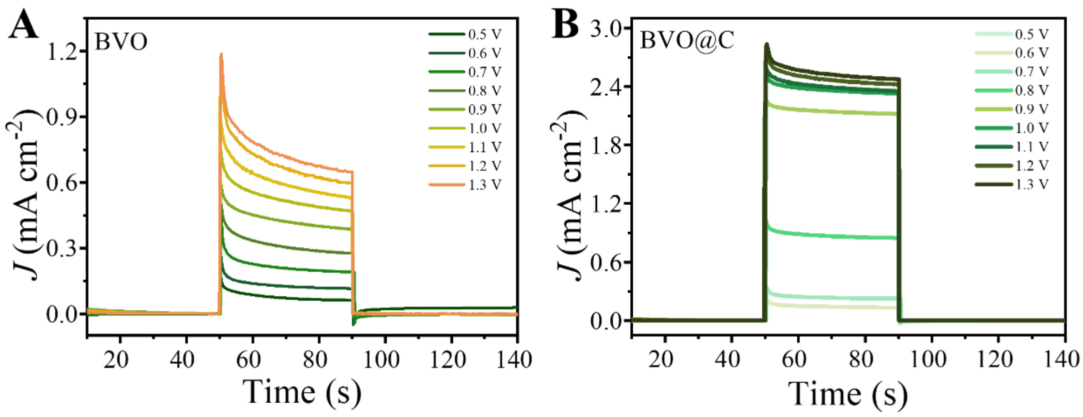

Figure S12. Light-switched $J-t$ curves of (A) BVO and (B) BVO@C photoanodes at different applied potentials versus RHE.

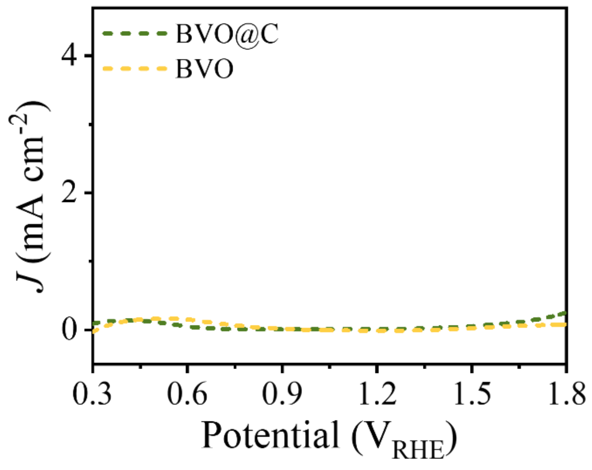

Figure S13. LSV curves of WOR under dark field for BVO and BVO@C photoanodes.

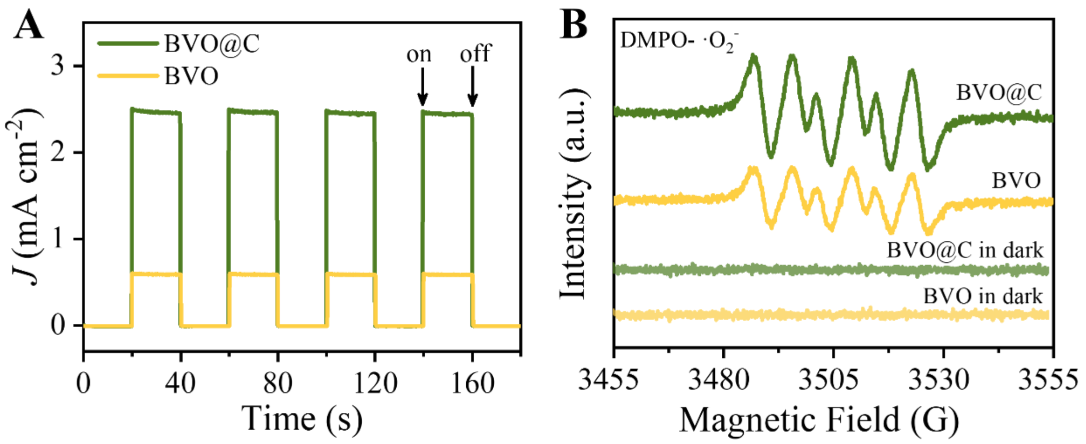

Figure S14. Light-switched $J-t$ curves at 1.23 V_{RHE} and EPR-electron capture curves for BVO and BVO@C photoanodes.

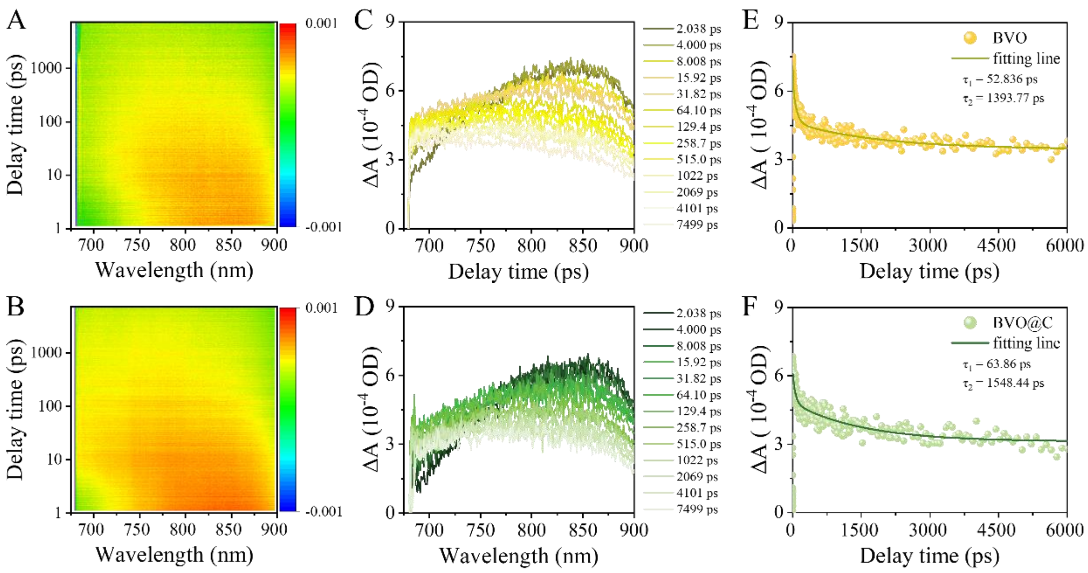

Figure S15. (A, B) Pseudo-colored TAS spectra of TAS spectra without voltage, (C, D) TAS spectra with different delay times, and (E, F) fitting curves of TAS decay dynamics of BVO and BVO@C photoanodes.

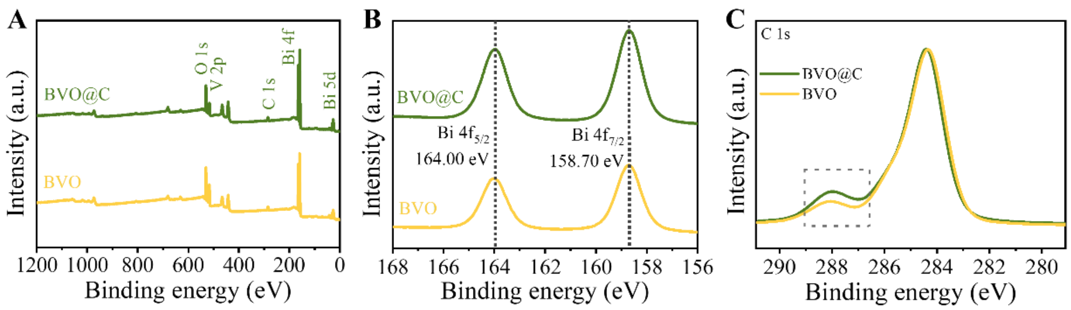

Figure S16. (A) XPS survey spectrum, and (B, C) high-resolution profiles of Bi 4f and C 1s for BVO@C and BVO photoanodes.

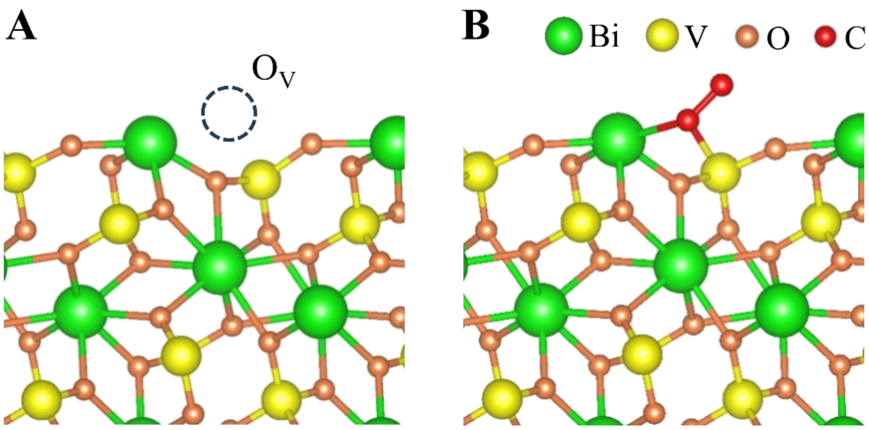

Figure S17. Structural models with oxygen defective surface and passivated surface for DFT calculations.

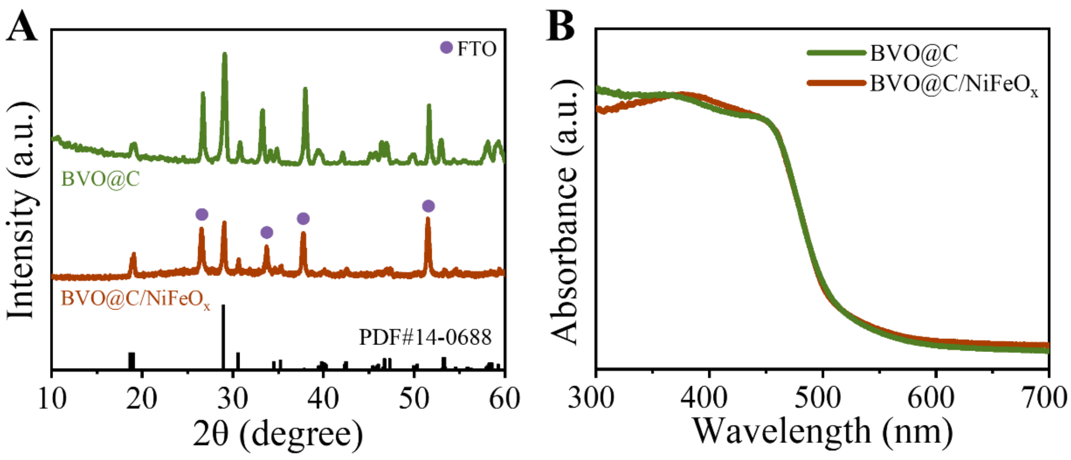

Figure S18. XRD patterns and UV-vis absorbance spectra of BVO@C and BVO@C/$NiFeO_x$ photoanodes.

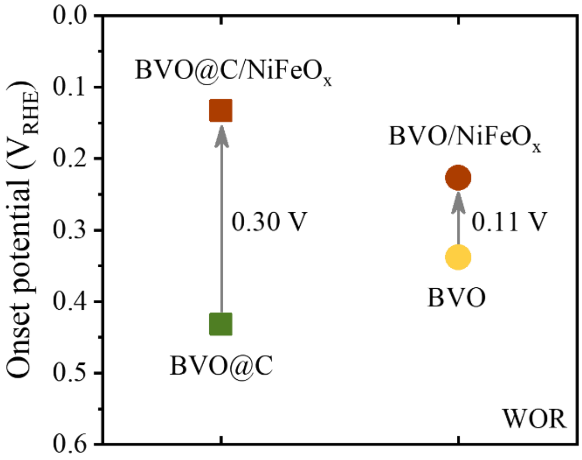

Figure S19. Cathodic offset values of onset potentials for BVO and BVO@C after the decoration of $NiFeO_x$ cocatalyst.

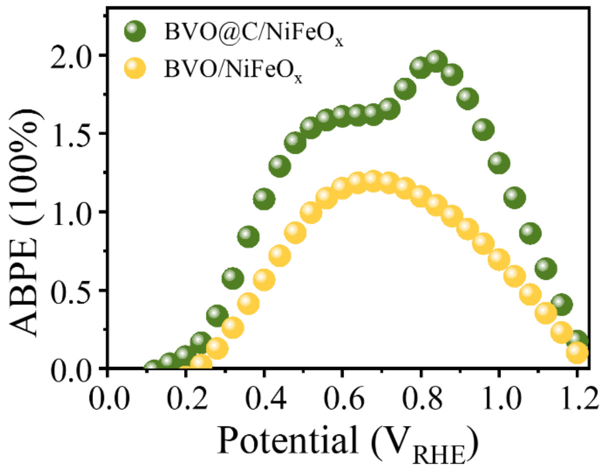

Figure S20. ABPE spectra of BVO@$NiFeO_x$ and BVO@C/$NiFeO_x$ photoanodes.

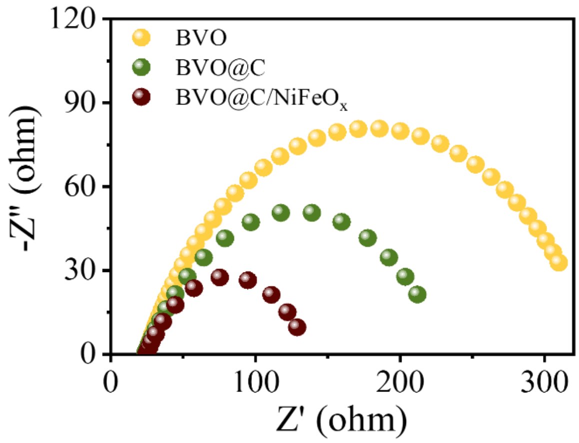

Figure S21. EIS Nyquist plots of BVO, BVO@C and BVO@C/$NiFeO_x$ recorded at a constant potential of 0.83 V_{RHE} under 1 sun illumination.

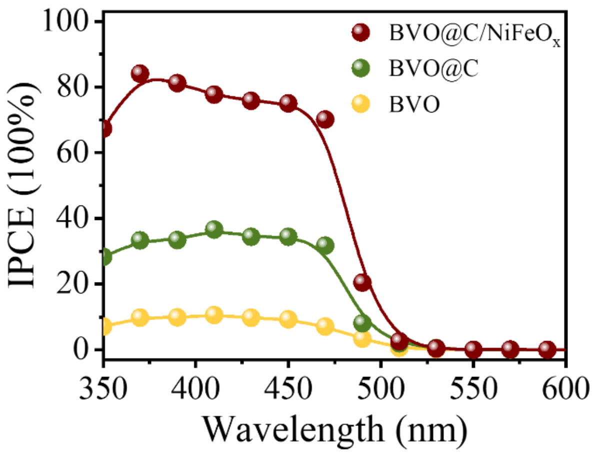

Figure S22. IPCE spectra of BVO, BVO@C and BVO@C/NiFeO$_x$ electrodes at 0.83 V_{RHE} with specific single-wavelength filters.

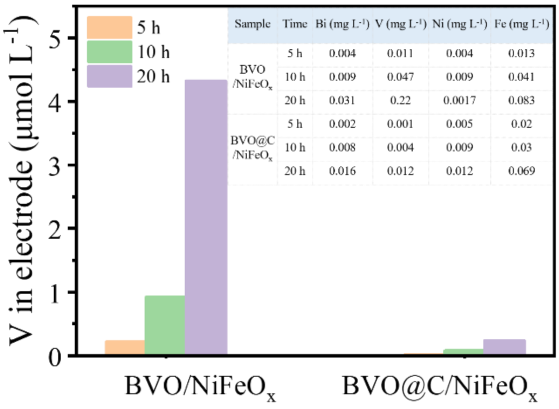

Sample	Time	Bi (mg L^{-1})	V (mg L^{-1})	Ni (mg L^{-1})	Fe (mg L^{-1})
BVO/$NiFeO_x$	5 h	0.004	0.011	0.004	0.013
	10 h	0.009	0.047	0.009	0.041
	20 h	0.031	0.22	0.0017	0.083
BVO@C/$NiFeO_x$	5 h	0.002	0.001	0.005	0.02
	10 h	0.008	0.004	0.009	0.03
	20 h	0.016	0.012	0.012	0.069

Figure S23. In-situ ICP-MS spectra during stability tests for BVO@$NiFeO_x$ and BVO@C/$NiFeO_x$ photoanodes

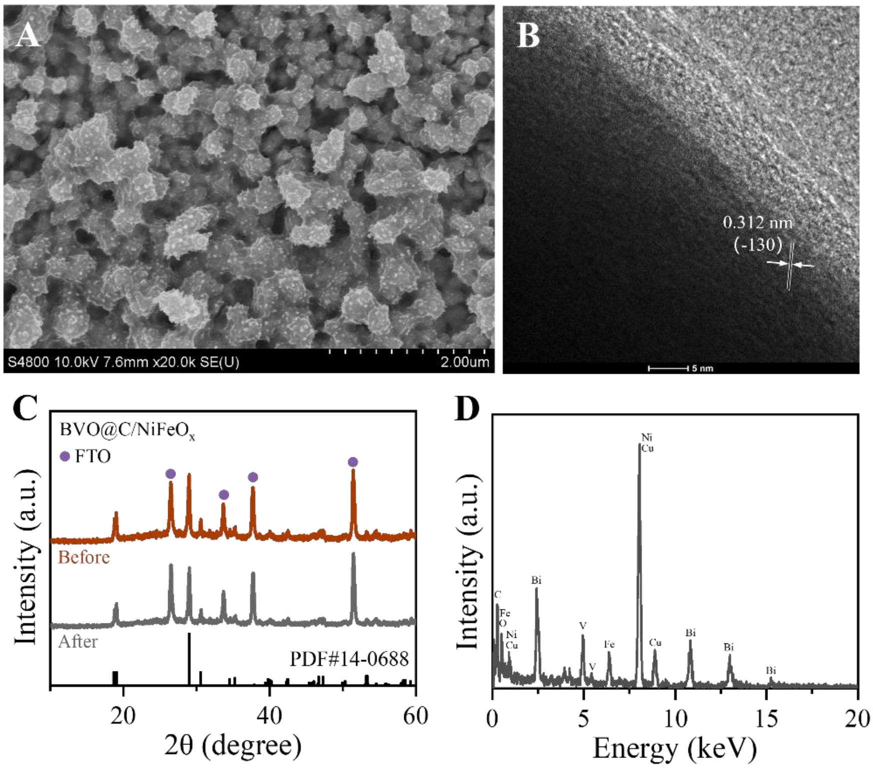

Figure S24. (A) SEM image, (B) TEM image, (C) XRD pattern and (D) EDS spectrum of BVO@C/NiFeOx photoanodes after stability test.

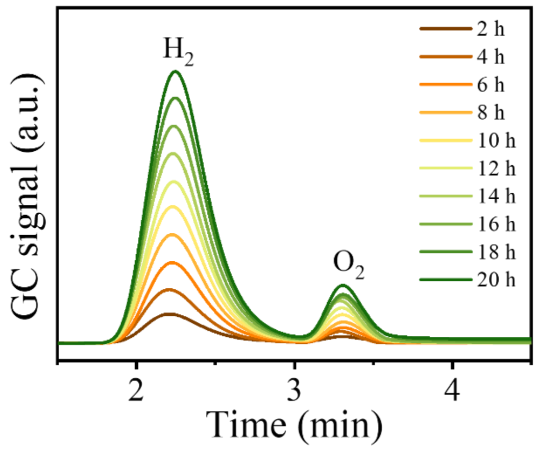

Figure S25. Time-dependent GC signals of gas amounts evolved over BVO@C/$NiFeO_x$ cell.

Table S1. Performance comparison of BVO@C/$NiFeO_x$ photoanode in this work and other BVO-based photoanodes reported previously in literatures.

Electrode	Cocatalyst	Performance (@1.23 V vs RHE)	Separation efficiency (%)	Transfer efficiency (%)	ABPE(%)	Stability test time, potential	Reference
BVO@C (1×1 cm^2)	$NiFeO_x$	5.9 mA cm^{-2} 0.5 M KBi (pH 9.3)	96.0	96.4	1.96% at 0.83 V_{RHE}	120 h, 1.23 V_{RHE}	This work
$BiVO_4$	CoNi-MOF	3.2 mA cm^{-2} 0.5 M Na_2SO_4(pH 7)	52.3	66.3	0.65% at 0.9 V_{RHE}	3 h, 1.23 V_{RHE}	Appl. Catal. B-Environ. 2020, 266, 118513
$BiVO_4$	NiFe-MOFs	4.61 mA cm^{-2} 0.5 M KBi (pH 9.5)	83	70	1.81% at 0.64 V_{RHE}	3 h, 1.23 V_{RHE}	Chem. Eng. J. 2022, 433, 133592
$BiVO_4$	$FeCoO_x$	4.82 mA cm^{-2} 1 M KBi (pH 9.5)	No	90	1.19% at 0.7 V_{RHE}	10 h, 1.23 V_{RHE}	Adv. Funct. Mater. 2018, 28, 1802685
$BiVO_4$	Co-silicate	5 mA cm^{-2} 0.5 M KBi (pH 9.5)	91	86	1.55% at 0.65 V_{RHE}	20 h, 1.23 V_{RHE}	Appl. Catal. B-Environ. 2020, 277, 119189
$BiVO_4$	$NiFeO_x$	5.54 mA cm^{-2} 1 M KBi (pH 9.5)	98.2	92.2	1.85% at 0.67 V_{RHE}	100 h, 1.23V_{RHE}	Adv. Mater. 2020, 32, 2001385
$BiVO_4$	Co-Pi	6.1 mA cm^{-2} 0.5 M PBS (pH 7)	71	96	No	6 h, 0.6 V_{RHE}	Energy Environ. Sci. 2018, 11, 1299
$BiVO_4$	N:$NiFeO_x$	6.4 mA cm^{-2} 0.5 M KBi (pH 9.5)	No	88	1.9% at 0.73 V_{RHE}	5 h, 1.23 V_{RHE}	Nat. Commun. 2021, 12, 6969
$BiVO_4$	$FeNiPO_x$	6.73 mA cm^{-2} 0.5 M KBi (pH 9.5)	No	97.4	2.48% at 0.71 VRHE	84 h, 1 V_{RHE}	Energy Environ. Sci. 2022, 15, 2867
$BiVO_4/Co_3O_4$	CoFe-LDH	2.98 mA cm^{-2} 0.5 M KPi (pH 7)	90	71	1.23% at 0.65 V_{RHE}	10 h, 0.6 V_{RHE}	Sol. RRL 2019, 3, 1900115
$BiVO_4/WO_3$	F:FeOOH	3.1 mA cm^{-2} 0.1 M PBS (pH 7.5)	66.8	74.4	0.57% at 0.89 V_{RHE}	3 h, 1.23 V_{RHE}	Appl. Catal. B-Environ. 2022,304, 120995
BP/$BiVO_4$	NiOOH	4.48 mA cm^{-2} 0.5 M KPi (pH 7.1)	95.8	90	No	60 h, 1.23 V_{RHE}	Nat. Commun. 2019, 10, 2001
$Bi_{1-x}VO_4$	Co-Bi	4.5 mA cm^{-2} 1M KBi (pH 9.5)	79	81.11	No	30 h, 1.23 V_{RHE}	Adv. Mater. 2022, 34, 2108178
Mo:$BiVO_4$	NiFe/tannic acid	5.1 mA cm^{-2} 0.5 M KBi (pH 8.5)	80	86	1.56% at 0.72 V_{RHE}	3 h, 1.23 V_{RHE}	ACS Energy Lett. 2018, 3, 1648
B:$BiVO_4$	$NiFeO_x$	5.22 mA cm^{-2} 0.5 M KBi (pH 9.5)	76.2	91.1	1.96% at 0.65 V_{RHE}	11 h, 0.8 V_{RHE}	ACS Sustainable Chem. Eng. 2021, 9, 8306
Mo:$BiVO_4$	CoAl-LDH	5.8 mA cm^{-2} 1 M KBi (pH 9.5)	87.3	98	1.87% at 0.73 V_{RHE}	1.39 h, 1 V_{RHE}	Chem. Eng. J. 2023,465,142893
MQD/$BiVO_4$	MoO_x/NiFeOOH	5.85 mA cm^{-2} 0.5 M KBi (pH 9.3)	No	91	2.43% at 0.58 V_{RHE}	100 h, 1.23 V_{RHE}	Angew. Chem. Int. Ed. 2022, 134, e202200946
